# Graves' Disease Presenting as Refractory Panic Attacks: Diagnostic Clarification Through Thyroid Scintigraphy

**DOI:** 10.1002/ccr3.73055

**Published:** 2026-07-01

**Authors:** Mahsa Satari Gholami

**Affiliations:** ^1^ Cardiovascular Nursing Research Center Rajaie Cardiovascular Institute Tehran Iran

**Keywords:** diagnostic challenge, graves' disease, neuropsychiatric symptoms, panic attacks, thyroid scintigraphy

## Abstract

Graves' disease may rarely present with predominant neuropsychiatric symptoms, such as severe, treatment‐resistant panic attacks, leading to misdiagnosis as a primary psychiatric disorder. We describe a 30‐year‐old woman with refractory panic attacks and night terrors who was initially treated with anxiolytics and antidepressants. Despite fluctuating thyroid function test results, which raised diagnostic uncertainty between evolving hyperthyroidism and thyroiditis, the presence of ophthalmopathy and autonomic hyperactivity prompted further evaluation. Thyroid scintigraphy demonstrated diffuse uptake consistent with Graves' disease and effectively excluded thyroiditis, allowing prompt initiation of antithyroid therapy and propranolol. Following treatment, the patient showed significant clinical improvement with resolution of autonomic symptoms. This case highlights the diagnostic challenge of atypical Graves' disease presentations and emphasizes the importance of considering endocrine causes in refractory neuropsychiatric symptoms. Functional imaging, alongside careful clinical assessment, may help resolve clinical–biochemical discrepancies and support timely diagnosis in selected patients with atypical presentations.

## Key Clinical Message

Graves' disease may initially present with challenging neuropsychiatric symptoms, such as panic attacks, even without clear signs of hyperthyroidism in blood tests. Clinicians should be mindful of endocrine issues and consider nuclear thyroid scanning when symptoms and lab results don't align to prevent delays in psychiatric care.

## 1 Introduction

Graves' disease (GD) is the most common cause of hyperthyroidism globally, characterized by an autoimmune response that produces stimulatory antibodies targeting the thyroid‐stimulating hormone receptor [1]. The conventional clinical presentation characterized by tachycardia, unintentional weight loss, heat intolerance, and tremors is widely acknowledged; however, the phenotypic diversity of Graves' disease is notably varied [2].

A significant diagnostic dilemma occurs when severe mental symptoms, particularly refractory panic attacks and widespread anxiety, present as the major or initial clinical manifestations [3]. In emergency and triage environments, when the feeling of “impending doom” characterizes the patient's experience, therapeutic attention may prematurely divert to psychotropic therapies, thereby masking the underlying endocrine disease and postponing final therapy [4]. Although serum thyrotropin and thyroid hormone measurements are highly sensitive for detecting hyperthyroidism, fluctuating biochemical results may occasionally create diagnostic uncertainty regarding the underlying etiology. In such situations, differentiation between Graves' disease and thyroiditis may require additional diagnostic evaluation. Functional imaging with thyroid scintigraphy can help establish the diagnosis by demonstrating increased uptake in Graves' disease and reduced uptake in destructive thyroiditis [5]. Furthermore, the presence of Graves' ophthalmopathy, even in the absence of overt biochemical thyrotoxicosis, necessitates a high index of suspicion. In diagnostically challenging cases where clinical findings and biochemical results are discordant, thyroid scintigraphy can serve as a valuable adjunctive diagnostic tool. By assessing radionuclide uptake patterns, scintigraphy may help differentiate Graves' disease from other causes of thyrotoxicosis, particularly when the diagnosis remains uncertain. Current diagnostic approaches to Graves' disease rely primarily on biochemical testing and thyroid receptor antibody (TRAb) assays. However, additional diagnostic modalities such as thyroid scintigraphy may be helpful when the clinical presentation and laboratory findings are not fully concordant or when the underlying etiology of thyrotoxicosis remains uncertain [6, 7]. By quantifying the global and focal radioisotope uptake, scintigraphy can confirm thyroid hyperfunction and differentiate GD from other forms of thyrotoxicosis, even when serological markers remain equivocal [7, 8].

In this report, we present a complex case of a patient with severe, treatment‐resistant panic attacks and mild ophthalmopathy, whose diagnosis of Graves' disease was initially obscured by non‐confirmatory thyroid function tests but was ultimately established through the strategic use of thyroid scintigraphy.

## 2 Case History/Examination

A 30‐year‐old female presented with a complex array of symptoms including progressive dyspnea, palpitations, muscle spasms, and multisite paresthesia (hands, feet, and scalp). The patient also reported chronic headaches, insomnia, and severe nocturnal agitation characterized by waking up screaming (nocturnal panic attacks). Physical examination revealed mild bilateral exophthalmos (which was observed and diagnosed during clinical examination), fine hand tremors, and persistent restlessness. Due to the predominance of neuropsychiatric features, the patient had initially been evaluated and treated by multiple healthcare providers for presumed panic and anxiety‐related symptoms prior to the diagnosis of Graves' disease. Before the conclusive diagnosis, the patient received treatment based on the presumption of a primary anxiety disorder, utilizing anxiolytic and antidepressant drugs such as alprazolam, venlafaxine, and trazodone.

In addition, he reported no history of smoking, drug use, or recreational drug use. He also denied a family history of autoimmune thyroid disease. Vital signs were generally stable, with the patient reporting palpitations and a heart rate of approximately 110 beats per minute. The patient's blood pressure was within normal limits and had not changed. The patient's weight had decreased by approximately 2.5 kg over the previous months.

Given the combination of chronic headaches, multisite paresthesia involving the hands, feet, and scalp, tremors, and prominent nocturnal agitation, neurological evaluation was pursued to exclude alternative central nervous system disorders. Although the clinical suspicion for an infectious or inflammatory CNS process was low, lumbar puncture was performed as part of the diagnostic workup to exclude conditions such as central nervous system infection, inflammatory disorders, or demyelinating disease. The cerebrospinal fluid (CSF) analysis, including opening pressure, cell count, protein, glucose, and cultures, yielded unremarkable results. Comprehensive laboratory screening, including hematological and biochemical parameters, was unremarkable except for mild iron deficiency anemia [Hemoglobin: 12 g/dL (12–16 g/dL), Ferritin: 10 ng/mL (13–150 ng/mL)]. Mild iron deficiency was identified during the diagnostic workup because iron deficiency may contribute to nonspecific neurological symptoms such as paresthesia, fatigue, and impaired well‐being. However, the degree of deficiency observed in this patient was insufficient to account for the severity of the autonomic and neuropsychiatric manifestations. Accordingly, iron and folic acid supplementation was initiated. The diagnostic challenge was compounded by fluctuating thyroid function tests (TFTs) performed at three‐day intervals. Initial results showed overt hyperthyroidism (TSH: 0.22 μIU/mL, T3: 3.1 nmol/L, T4: 160 nmol/L), while subsequent tests fluctuated between euthyroid and mildly thyrotoxic states (Table 1). The fluctuating thyroid function tests over the subsequent days, together with the prolonged diagnostic course and predominant neuropsychiatric presentation, created uncertainty regarding the underlying etiology of thyrotoxicosis. At the time of evaluation, alternative diagnoses including thyroiditis (silent or subacute thyroiditis), toxic adenoma, toxic multinodular goiter, factitious thyrotoxicosis, and early Graves' disease were considered. Because a rapid and definitive etiological diagnosis was required to guide treatment decisions, thyroid scintigraphy was performed. Imaging including abdominal/pelvic CT and chest radiography revealed no abnormalities. Because serial thyroid function tests (TSH, T3, and T4) showed fluctuating results that were inconsistent with the severity of the patient's clinical presentation, and because prompt diagnostic clarification was required to guide management, the nuclear thyroid scan was performed for definitive functional assessment.

**TABLE 1:** Fluctuating thyroid function tests over a 7‐day period.

Parameter	Initial (Day 1)	Day 4	Day 7	Reference range
TSH (μIU/mL)	0.22 (Low)	0.42 (Normal)	1.59 (Normal)	0.35–4.94
T4 (nmol/L)	160 (High)	130 (Normal)	154 (High)	62.68–150.85
T3 (nmol/L)	3.1 (High)	1.5 (Normal)	1.26 (Normal)	0.89–2.42

Thyroid ultrasonography demonstrated mild diffuse enlargement of both thyroid lobes with otherwise unremarkable sonographic findings. Thyroid echotexture and echogenicity were preserved, with no evidence of focal nodules, cystic lesions, calcifications, or other structural abnormalities. No significant cervical lymphadenopathy was identified. Although the ultrasonographic findings were nonspecific, the diffuse thyroid enlargement supported further evaluation for underlying thyroid pathology.

The patient was evaluated by multiple healthcare providers over approximately 35 days and was initially treated for presumed psychiatric disorders before thyroid scintigraphy (nuclear thyroid scan) established the definitive diagnosis of Graves' disease.

**FIGURE 1:**
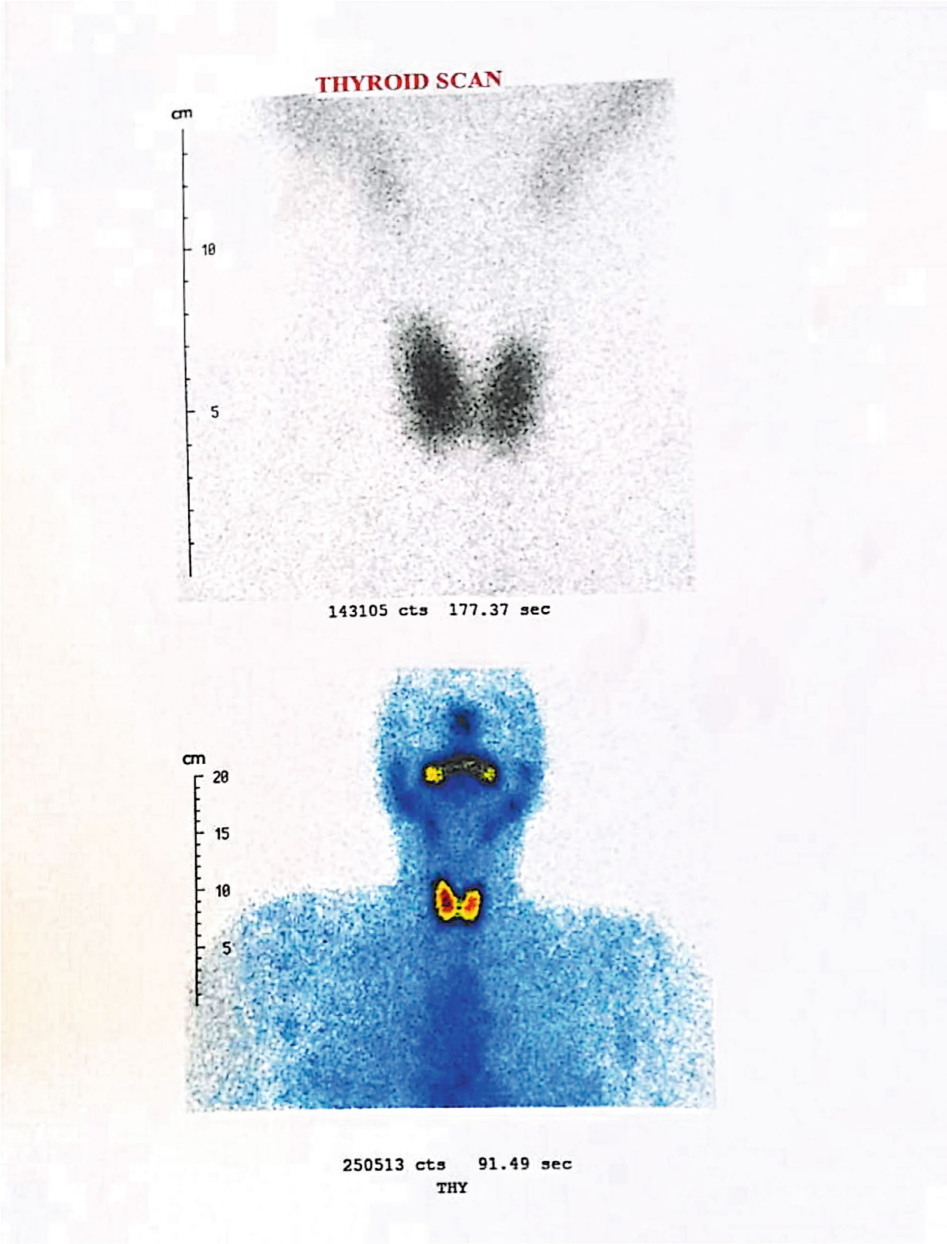
Nuclear scan image of the patient.

Due to the clinical–biochemical discrepancy, a 5 mCi 99mTc‐04 pertechnetate thyroid scintigraphy was performed. The scan revealed a mild‐to‐moderately enlarged thyroid gland with uniformly increased radiotracer uptake throughout both lobes, consistent with Graves' disease (Figure 1).

Methimazole was initially prescribed at a dose of 5 mg three times daily and was subsequently tapered based on serial thyroid function test results. Over the course of approximately 18 months, the dose was gradually reduced, and the patient is currently taking methimazole approximately twice weekly. Thyroid function tests are monitored every 3 months, and the patient remains under regular physician supervision.

Propranolol was initiated at a dose of 10 mg twice daily. As the patient's symptoms, including palpitations, improved, the dose was progressively reduced to the point that it was taken only as needed for palpitations. The patient has now discontinued propranolol completely.

During this period (18 months), the aforementioned symptoms have gradually decreased to the point where the patient now has none of the symptoms, but he is still under the supervision of a specialist physician and periodic (quarterly) check‐ups and tests (TFTs) are performed.

## 3 Differential Diagnosis

Given the patient's predominant neuropsychiatric manifestations, autonomic symptoms, and fluctuating laboratory findings, several differential diagnoses were considered. Primary psychiatric disorders, including panic disorder and generalized anxiety disorder, were initially suspected due to severe panic attacks, insomnia, and nocturnal agitation. However, the persistence of autonomic symptoms, tremors, and ophthalmic findings despite psychiatric treatment prompted further investigation for an underlying medical cause.

Neurological conditions such as multiple sclerosis, meningitis, peripheral neuropathy, and other central nervous system infectious or inflammatory disorders were considered in light of multisite paresthesia, tremors, chronic headaches, and night terrors. Cerebrospinal fluid analysis, including cell count, protein, glucose, and cultures, was unremarkable, making infectious and inflammatory central nervous system disorders less likely. In addition, the absence of focal neurological deficits and the lack of supportive clinical findings reduced the likelihood of demyelinating disorders such as multiple sclerosis.

Metabolic and endocrine disorders, including pheochromocytoma, hypoglycemia, electrolyte imbalances, thyroiditis, toxic multinodular goiter, factitious thyrotoxicosis, and thyroid dysfunction, were also evaluated as potential causes of autonomic hyperactivity and thyrotoxic symptoms. Routine biochemical investigations did not reveal evidence of hypoglycemia or electrolyte abnormalities. Although thyroiditis was considered because of the fluctuating thyroid function test results, the diffuse and homogeneous radiotracer uptake observed on thyroid scintigraphy was inconsistent with destructive thyroiditis. Toxic multinodular goiter was considered less likely because scintigraphy demonstrated diffuse rather than focal or nodular uptake. Factitious thyrotoxicosis was also considered unlikely given the absence of exogenous thyroid hormone use and the presence of increased radionuclide uptake on thyroid scintigraphy.

Iron deficiency‐related neuropathy was considered due to low ferritin levels but was deemed insufficient to explain the severity and broad spectrum of the patient's neuropsychiatric and autonomic symptoms. Ultimately, the presence of ophthalmopathy, tremors, persistent autonomic symptoms, and diffusely increased radiotracer uptake on thyroid scintigraphy supported the diagnosis of Graves' disease despite inconsistent biochemical findings.

## 4 Conclusion and Results (Outcome and Follow‐Up)

Graves' disease can occasionally present with predominant neuropsychiatric symptoms, including treatment‐resistant panic attacks and nocturnal anxiety, which may mimic primary psychiatric disorders and contribute to diagnostic delay. Clinical–biochemical discrepancies, as observed in fluctuating thyroid function tests, highlight the limitations of relying solely on serological markers in atypical cases. Functional imaging, such as thyroid scintigraphy, may serve as a valuable diagnostic adjunct when clinical suspicion remains high despite inconclusive laboratory findings. This case underscores the importance of considering endocrine etiologies in patients with refractory psychiatric symptoms and supports a multimodal diagnostic approach to facilitate timely diagnosis and appropriate management.

## 5 Discussion

The present case highlights the profound diagnostic challenges inherent in the “clinical masquerade” of Graves' disease as a primary psychiatric disorder. The predominant neuropsychiatric features in our patient, specifically the refractory panic attacks and night terrors, led to an initial misdiagnosis and the suboptimal initiation of antidepressant therapy.

A distinctive feature of our case was the observed clinical biochemical discrepancy; while the patient exhibited severe autonomic symptoms and ophthalmopathy, the thyroid function tests fluctuated between overt hyperthyroidism and euthyroid states. Such variability underscores the limitations of relying solely on serological markers in the early stages of autoimmune thyroiditis. In these diagnostic deadlocks, thyroid scintigraphy proved to be an indispensable tool, providing a definitive quantification of global radiotracer uptake and confirming the underlying Graves' pathology despite equivocal laboratory results.

The neuropsychiatric manifestations observed in Graves' disease are thought to arise from several interacting pathophysiological mechanisms. Excess thyroid hormones enhance central and peripheral sensitivity to catecholamines by increasing β‐adrenergic receptor expression and activity, resulting in palpitations, tremor, hyperarousal, and anxiety‐like symptoms that may closely resemble panic disorder. In addition, thyroid hormones exert direct effects on the central nervous system, influencing neurotransmitter systems including serotonin, dopamine, and norepinephrine, which are involved in mood regulation and emotional processing. Autonomic dysregulation, characterized by sympathetic overactivity and impaired parasympathetic modulation, has also been implicated in the development of anxiety, sleep disturbances, and stress‐related symptoms in hyperthyroid patients [3].

In a 2021 case report published in BioPsychoSocial Medicine, Yasuda and colleagues described a 34‐year‐old woman whose evolving symptoms of Graves' disease were initially misinterpreted as panic attacks due to pre‐existing panic and bipolar diagnoses, illustrating how overlapping clinical features can delay correct identification of underlying hyperthyroidism. Despite presenting with weight loss, palpitations, nausea, restlessness, and proptosis, the diagnosis was deferred until thyroid function tests became available, at which point she was found to be in thyroid storm and promptly treated with steroids, antithyroid drugs, and radioactive iodine, resulting in recovery. The authors emphasize the importance of considering severe hyperthyroidism in the differential diagnosis of panic symptoms, even in psychiatric populations, to prevent life‐threatening endocrine emergencies [4].

Similar to our case, this report highlights how Graves' disease may initially masquerade as panic disorder, leading to delayed diagnosis; however, our case further underscores the diagnostic value of thyroid scintigraphy in resolving clinical–biochemical discordance before progression to severe complications.

Yamaguchi et al. reported a 38‐year‐old female who presented with bilateral ptosis, double vision, tremors, paresthesia, weight loss, palpitations, and a neck swelling, ultimately diagnosed with Graves' disease complicated by ocular myasthenia. Initial evaluation revealed hyperthyroidism with suppressed TSH and elevated T3/T4, diffuse goiter on ultrasonography, and increased tracer uptake on technetium thyroid scan. Neurological studies, including repetitive nerve stimulation, along with a positive neostigmine test, confirmed ocular myasthenia. The patient was treated with carbimazole and pyridostigmine, resulting in significant improvement of thyrotoxic symptoms, while mild ptosis and residual ophthalmoplegia persisted on follow‐up. This case highlights how Graves' disease can present predominantly with neuro‐ophthalmic and neuromuscular symptoms, which may mimic primary neurological or psychiatric disorders, leading to potential diagnostic delays. Similar to our report, it emphasizes the importance of considering thyroid dysfunction in patients presenting with atypical or refractory neuropsychiatric symptoms, and using targeted investigations such as thyroid scans to reach a definitive diagnosis [9].

Beyond establishing the diagnosis and correcting the underlying endocrine abnormality, patients with prominent neuropsychiatric manifestations may benefit from supportive multidisciplinary management strategies. Neuropsychiatric manifestations associated with systemic illnesses such as Graves' disease may require supportive multidisciplinary management strategies in addition to correction of the underlying endocrine abnormality. Recent literature suggests that exercise‐based and rehabilitative interventions may contribute to improvement in anxiety, stress regulation, and overall psychological well‐being through modulation of neurobiological pathways and autonomic function [8]. Such approaches may complement pharmacological treatment and facilitate recovery while thyroid function is being stabilized [10].

A limitation of this case is that thyroid autoantibody testing, including TSH receptor antibodies (TRAb), was not performed during the diagnostic evaluation. This investigation could have provided additional serological support for the diagnosis of Graves' disease. The treating physician prioritized rapid diagnostic clarification because of the patient's prolonged symptomatic course, marked autonomic and neuropsychiatric manifestations, and fluctuating thyroid function test results. In this clinical context, thyroid scintigraphy was selected as a readily available modality capable of providing prompt functional assessment and facilitating differentiation between Graves' disease and other causes of thyrotoxicosis, particularly thyroiditis.

This case reinforces the necessity of maintaining a high index of suspicion for endocrine etiologies in all patients presenting with treatment‐resistant anxiety, advocating for a multi‐modal diagnostic approach that integrates functional imaging when biochemical data remain inconclusive.

## Author Contributions

**Mahsa Satari Gholami:** data curation, methodology, project administration, supervision, validation, writing – original draft, writing – review and editing.

## Funding

The author has nothing to report.

## Consent

Written informed consent was obtained from the patient for the publication of this case report and any accompanying images. The patient's identity has been protected, and all personal identifiers have been removed to ensure anonymity.

## Data Availability

The data that support the findings of this study are available from the corresponding author upon reasonable request.
